# Prioritization of head and neck cancer patient care during the COVID-19 pandemic: a retrospective cohort study

**DOI:** 10.1186/s40463-023-00625-w

**Published:** 2023-02-14

**Authors:** Samuel S. Psycharis, Samer Salameh, Sena Turkdogan, Saad Razzaq, Kevin Zhao, Marco A. Mascarella, Keith Richardson, Alex M. Mlynarek, Michael P. Hier, Nader Sadeghi

**Affiliations:** 1 Faculty of Dental Medicine and Oral Health Sciences, McGill University, Montreal, QC Canada; 2 Faculty of Medicine and Health Sciences, McGill University, Montreal, QC Canada; 3 Department of Otolaryngology Head and Neck Surgery, McGill University Health Centre, 1001 Decarie Blvd, Montreal, QC H4A 3J1 Canada; 4 Department of Otolaryngology Head and Neck Surgery, Jewish General Hospital, Montreal, QC Canada; 5 Research Institute, McGill University Health Centre, Montreal, Canada

**Keywords:** COVID-19, SARS-CoV-2, Coronavirus, Head and neck cancer, Otolaryngology

## Abstract

**Background:**

The COVID-19 pandemic placed considerable strain on the healthcare system, leading to the re-allocation of resources and implementation of new practice guidelines. The objective of this study is to assess the impact of COVID-19 guideline modifications on head and neck cancer (HNC) care at two tertiary care centers in Canada.

**Methods:**

A retrospective cohort study was conducted. HNC patients seen at two tertiary care centers before and after the onset of the COVID-19 pandemic (pre-pandemic: July 1st, 2019, to February 29th, 2020; pandemic: March 1st, 2020, to October 31st, 2020) were included. The pre-pandemic and pandemic cohorts were compared according to patient and tumor characteristics, duration of HNC workup, and treatment type and duration. Mean differences in cancer care wait times, including time to diagnosis, tumor board, and treatment as well as total treatment package time and postoperative hospital stay were compared between cohorts. Univariate and multivariate analyses were used to compare characteristics and outcomes between cohorts.

**Results:**

Pre-pandemic (n = 132) and pandemic (n = 133) patients did not differ significantly in sex, age, habits, or tumor characteristics. The percentage of patients who received surgery only, chemo/radiotherapy (CXRT) only, and surgery plus adjuvant CXRT did not differ significantly between cohorts. Pandemic patients experienced a significant time reduction compared to pre-pandemic patients with regards to the date first seen by a HNC service until start of treatment ($\overline{x}$ = 48.7 and 76.6 days respectively; *p* = .0001), the date first seen by a HNC service until first presentation at tumor board ($\overline{x}$ = 25.1 and 38 days respectively; *p* = .001), mean total package time for patients who received surgery only ($\overline{x}$ = 3.7 and 9.0 days respectively; *p* = .017), and mean total package time for patients who received surgery plus adjuvant CXRT ($\overline{x}$ = 80.2 and 112.7 days respectively; *p* = .035).

**Conclusion:**

The time to treatment was significantly reduced during the COVID-19 pandemic as compared to pre-pandemic. This transparent model of patient-centered operative-room prioritization can serve as a model for improving resource allocation and efficiency of HNC care during emergency and non-emergency scenarios.

## Background

Since its emergence, the COVID-19 pandemic has placed a significant strain on healthcare systems worldwide. Essential health services and resources were diverted towards caring for COVID-19 infected patients, and additional hospital prioritization measures were taken for hospital resource allocation. New guidelines pertaining to triage and delivery of cancer care were implemented for the management of Head and Neck cancer (HNC) patients [1–7]. Studies suggesting that cancer patients are at higher risk of contracting the SARS-CoV-2 virus and experiencing poorer COVID-19 outcomes further validated the need for HNC patient care guideline modifications [8, 9]. However, a reduction in healthcare personnel and overall time in the operating room (OR) may have led to delaying surgeries for early and low-risk HNC cases in favor of serial monitoring or non-surgical options, with intervention only considered for progression [1]. Despite the prioritization of advanced HNC, alternative treatments such as radiotherapy or chemoradiotherapy, were recommended for these patients when OR time could not be feasible [1].

Overall, the beginning of the pandemic witnessed a sizable shift toward telemedicine consultations, when possible, for non-urgent cases in order to minimize the risk of virus transmission. However, while telemedicine may have circumstantially improved accessibility to healthcare and reduced risk of infection, it still provided challenges with respect to patient care and logistics. Physicians found it more difficult to virtually diagnose patients due to the lack of physical examination or in-person communication, including body language [10, 11]. Furthermore, telemedicine may have reduced accessibility for vulnerable populations, as well as disrupted specialty referral patterns [11]. Accordingly, reduced specialty referrals, prolonged triage periods, and treatment and/or operative delays may have had detrimental effects on cancer patient outcomes [12–14]. A meta-analysis by Hanna et al. suggested that a 4-week delay in cancer surgery increases mortality rate by approximately 6–8% across several major cancer sites including the head and neck [15]. Based on this estimate, recent models predict that disruptions in cancer care during the pandemic could lead to 21,247 (2%) more cancer deaths in Canada in 2020–2030, assuming treatment capacity recovers to 2019 pre-pandemic levels in 2021 [16]. Given the rapid, qualitative, and heterogeneous manner in which guideline modifications were made during the pandemic, it is critical to gather quantitative evidence in order to examine the impact of these modifications on HNC patient care [17].

The objective of this study is to quantify the impact of COVID-19 pandemic guideline modifications on HNC patient care at two major academic tertiary care centers in Montreal, Quebec between March 1st and October 31st, in the year 2020. In particular, we looked at the effect of guideline modifications on Otolaryngology—Head and Neck Surgery (OTL-HNS) referrals, telemedicine visits, triage, treatment/operative delays, and cancer stage at presentation and treatment/operation. We are interested in answering the following questions:

1. How did HNC patient care during the pandemic differ from HNC patient care before the pandemic?
2. Did guideline modifications result in a reduced number of OTL-HNS referrals, increased reliance on telemedicine visits, prolonged triage times, and delays in treatments/operations?

## Methods

### Study design and population

This retrospective cohort study examined all HNC patients who were seen between July 1st, 2019, and October 31st, 2020, by the OTL-HNS Departments of two major academic tertiary care centers in Montreal, Quebec, Canada. Inclusion criteria was any adult patient over the age of 18 who presented for workup and diagnosis of a new cancer, or findings of recurrence for their pre-existing cancer. A comprehensive chart review of patients identified using the Cancer Diagnosis and Treatment Committees database of the McGill University Health Centre Cancer Registry for the months of July 2019 to October 2020 inclusively was conducted (Appendix, Table 6). These months were selected based on the World Health Organization's decision to officially declare COVID-19 a global pandemic in March 2020 and the simultaneous beginning of OTL-HNS guideline modifications imposed at the two academic tertiary care centers, with an equal duration of time immediately prior to March serving as the comparison.

### Exposure

Patients were separated into two groups based on the date of their first OTL-HNS consult (exposed/pandemic group: March 1st, 2020–October 31st, 2020; non-exposed/pre-pandemic group: July 1st, 2019–February 29th, 2020). Any patients that had five or more dates prior to the date of start of treatment (out of eight total) missing in their charts were excluded due to the uncertainty surrounding those cases (Appendix, Table 7).

### Covariates

Sociodemographic factors including patient sex, age, smoking and alcohol consumption status were collected given their impact on cancer risk. Patients were categorized as positive for smoking or alcohol consumption in their charts by experienced OTL-HNS clinicians based on current use or significant history of use (i.e. at least 10 pack-years for smoking). Tumor characteristics such as recurrence and tumor staging according to the American Joint Committee on Cancer (AJCC) 8th edition were also recorded since they would have influenced the work-up and treatment. With regards to treatment type, patients from both cohorts were divided into three categories: surgery only, chemotherapy and/or radiotherapy (CXRT) only, and surgery plus adjuvant CXRT. Lastly, we gathered information regarding telemedicine usage and treatment intent for their potential effects on the outcomes of this study.

### Outcomes

The primary outcome of interest was the mean wait time for patients from the first OTL-HNS visit to definitive treatment. Secondary outcomes included the mean wait time from first OTL-HNS visit to presentation at tumor boards, the mean wait time from the date of first biopsy to histopathological diagnosis, the mean length of postoperative hospital stay, and the mean total package time. Based on treatment category, total package time was defined as either the duration of hospital stay (surgery only), or from start to end date of CXRT (CXRT only), or from date of first surgery to end of adjuvant treatment (surgery plus adjuvant CXRT).

### Statistical analyses

Two-way independent t-tests assuming equal variance between the pre-pandemic and pandemic group were used to assess mean differences in wait time and package time. These tests were also used to compare continuous demographic variables. Statistically significant changes in wait time and package time were adjusted for covariates (age, tumor type, tumor site, tumor staging) using multivariate regression analysis. Categorical demographic data were analyzed by Pearson's chi-squared tests. Chi-squared tests were also used to compare the mean number of patients with various tumor sizes between groups according to the AJCC 8th edition. All statistical analyses were performed using commercially available software (﻿SPSS Statistics version 27.0 & R version 4.2.0).

## Results

After combining data from both hospital centers, a total of 292 adult HNC patients (147 pre-pandemic and 145 pandemic) were identified within the study period. Of those, 15 pre-pandemic and 12 pandemic patients were excluded, due to missing chart information, resulting in a total of 265 patients (132 pre-pandemic and 133 pandemic) who were included in the study. There was no significant difference in baseline characteristics found between the groups in terms of age at symptom onset, sex, alcohol and tobacco consumption. With respect to treatment, there were no significant differences in the number of patients within each treatment category (surgery only, CXRT only, and surgery plus adjuvant CXRT) and the number of patients that were treated with curative intent as opposed to palliation. However, there were non-significant trends showing reductions in the proportion of curative treatments (6% reduction; *p* = 0.102) and patients in the surgery only category (32% reduction; *p* = 0.125). Patient characteristics, including socio-demographics and treatment type are summarized in Table 1.

**Table 1:** Patient characteristics

	Pre-pandemic group (n = 132)	Pandemic group (n = 133)	p-value
Sociodemographics			
Sex, *male* (%)	91 (69)	99 (74)	0.321
Age, *mean years* (SD)	65.35 (13.87)	66.50 (13.99)	0.500
Smoking (%)	61 (46)	57 (43)	0.658
Alcohol use (%)	53 (40)	55 (41)	0.762
Treatments			
Curative-intent treatment (%)	121 (93.1)	114 (87.0)	0.102
Surgery only (%)	34 (30.6)	23 (21.5)	0.125
Chemotherapy/radiotherapy only (%)	38 (34.2)	42 (39.3)	0.442
Surgery + adjuvant therapy (%)	39 (35.1)	42 (39.3)	0.529

*Significant difference (p < .05)

Our patient cohorts did not demonstrate any significant differences in baseline tumor characteristics including new versus recurrent cases, tumor site, tumor type, and tumor AJCC staging (Tables 2 and 3). However, there was a trend in progressive cancer presentations in the pandemic group including more T3/T4 cases (18% increase; *p* = 0.432) and more patients presenting with metastasis (80% increase; *p* = 0.386). Compared to the pre-pandemic group, it was found that significantly more patients had their first encounter with the OTL-HNS Department via telemedicine (n = 7; *p* = 0.01), and there was a tendency for more patients to be seen by telemedicine during their first visit with any doctor for index symptoms (700% increase; *p* = 0.082) during the pandemic (Table 4).

**Table 2:** Tumor characteristics

	Pre-pandemic group (n = 132)	Pandemic group (n = 133)	p-value
Incident cancers (%)	105 (82)	102 (79)	0.529
Tumor site			
Thyroid (%)	8 (6.1)	6 (4.5)	0.573
Tonsil (%)	16 (12.1)	11 (8.3)	0.300
Base of tongue (%)	7 (5.3)	14 (10.5)	0.116
Parotid (%)	9 (6.8)	7 (5.3)	0.595
Cheek (%)	4 (3.0)	9 (6.8)	0.159
Other (%)	88 (66.7)	86 (64.7)	0.731
Tumor type			
SCC			
SCC NOS (%)	40 (30.5)	52 (33.1)	0.639
SCC p16 + (%)	27 (20.6)	29 (18.5)	0.648
cSCC (%)	15 (11.5)	11 (8.2)	0.375
EBV-associated (%)	4 (3.1)	3 (2.2)	0.679
Melanoma (%)	6 (4.6)	7 (5.2)	0.808
Other (%)	39 (29.8)	32 (23.9)	0.279

*Significant difference (p < .05)

*SCC* Squamous cell carcinoma, *cSCC* cutaneous SCC, *NOS* not otherwise specified, *EBV* Epstein Barr Virus

**Table 3:** Tumor staging

	Pre-pandemic group (n = 132)	Pandemic group (n = 133)	p-value
Tumor size			
T1/T2 (%)	50 (52.6)	47 (47.0)	0.432
T3/T4 (%)	45 (47.4)	53 (53.0)	0.432
Lymph nodes			
N0 (%)	42 (46.2)	48 (46.2)	0.465
N1 (%)	21 (23.1)	27 (26)	0.485
N2 (%)	20 (22)	24 (23.1)	0.805
N3 (%)	8 (8.8)	5 (4.8)	0.887
Metastasis			
M1 (%)	5 (5.1)	9 (8.1)	0.386

*Significant difference (p < .05)

**Table 4.** Telemedicine usage

	Pre-pandemic group (n = 132)	Pandemic Group (n = 133)	p-value
First doctor visits/referrals by telemedicine	1	7	0.082
First encounters with OTL-HNS by telemedicine	0	7	0.010*

*Significant difference (p < .05)

As described in Table 5, the mean wait time from the date first seen by the OTL-HNS Department until the date first presented at tumor board was significantly reduced in the pandemic group relative to the pre-pandemic group ($\overline{x}$ = 25.1 and 38 days respectively; *p* = 0.001). The same was true regarding the date first seen by the OTL-HNS Department until the start of treatment ($\overline{x}$ = 48.7 and 76.6 days respectively; *p* = 0.001), the mean total package time for the surgery only category ($\overline{x}$ = 3.7 and 9.0 days respectively; *p* = 0.017), and the mean total package time for the surgery plus adjuvant CXRT category ($\overline{x}$ = 80.2 and 112.7 days respectively; *p* = 0.035). Notably, the mean total package time for the CXRT only category was significantly increased in the pandemic group relative to the pre-pandemic group ($\overline{x}$ = 53.3 and 36.6 days respectively; *p* = 0.011).

**Table 5:** Mean wait times in HNC workup and treatment

	Preliminary analysis			Multivariate analysis		
	Pre-pandemic group	Pandemic group	p-value (No Adjustment)	p-value (Adjustment A)	p-value (Adjustment B)	p-value (Adjustment C)
Date first seen by OTL-HNS Department *until* date first presented at tumor board, *mean # days*	38	25.1	0.001*	0.743	0.0009*	-
Date first seen by OTL-HNS Department *until* start date of definitive treatment, *mean # days*	76.6	48.7	0.0001*	0.799	0.012*	-
Date first biopsied *until* date of histopathological diagnosis, *mean # days*	14.1	9.9	0.142	-	-	-
Length of postoperative hospital stay, *mean # days*^*1*^	6.4	5.9	0.358	-	-	-
Total package time (surgery + adjuvant radio/chemo), *mean # days*	112.7	80.2	0.035*	0.854	0.308	0.0001*
Total package time (surgery only), *mean # days*^*2*^	9.0	3.7	0.017*	0.156	0.031*	-
Total package time (radio/chemo only),* mean # days*	36.6	53.3	0.011*	0.403	0.079	0.046*

*Significant difference (p < .05)

^1^“Length of postoperative hospital stay” is defined as the length of admission for all patients who received surgery regardless of whether they received adjuvant treatment

^2^“Total package time (surgery only)” is defined as the length of admission for patients who received only surgery without adjuvant treatment

There were no significant changes between groups for the remaining time intervals gathered; however, the following non-significant trends were observed: The pandemic group experienced a trend towards less delays than the pre-pandemic group from the date onset of symptoms until start of treatment ($\overline{x}$ = 179.6 and 191.9 days respectively; *p* = 0.234), from date first seen by any doctor until start of treatment ($\overline{x}$ = 88 and 106.3 days respectively; *p* = 0.089), and from date first biopsied until date of histopathological diagnosis ($\overline{x}$ = 9.9 and 14.1 days respectively; *p* = 0.142). Conversely, the delay from the date first seen by any doctor until the date first seen by the OTL-HNS Department of any type (whether in-person or via telemedicine) was slightly but not significantly longer during the pandemic versus before the pandemic ($\overline{x}$ = 54.6 and 42.1 days respectively; *p* = 0.142). The length of postoperative stay was also relatively similar between groups ($\overline{x}$ = 5.9 days in the pandemic group, and 6.4 days in the pre-pandemic group; *p* = 0.358) (Table 5).

Multivariate regression analysis was performed on significant changes in wait times and total package times, whereby the results were adjusted for pertinent covariates (Table 5). Initially, when the results were adjusted for age, tumor site, tumor type, and tumor TNM staging (Adjustment A), none of the results were significant. Subsequently, the results were adjusted for all tumor characteristics (site, type, and staging) without age (Adjustment B): this yielded significance for date first seen by OTL-HNS to date first presented at tumor board (*p* = 0.0009), date first seen by OTL-HNS to start date of treatment (*p* = 0.012), and mean total package time for Surgery Only (*p* = 0.031). Since the mean total package times for surgery plus adjuvant CXRT and CXRT only categories were non-significant after Adjustment B, these results were partially adjusted for tumor characteristics (Adjustment C). The mean total package time for CXRT only was significant after adjusting for all tumor characteristics except metastasis (*p* = 0.046), and the mean total package time for surgery plus adjuvant CXRT was significant after adjusting for all tumor characteristics except tumor size and metastasis (*p* = 0.0001).

## Discussion

The COVID-19 pandemic had a profound impact on healthcare system guidelines worldwide. In particular, the early stages of the pandemic required a considerable proportion of healthcare personnel and equipment to be reallocated towards relieving the growing burden of caring for COVID-19 patients. A 2021 study on one tertiary-care center showed that during the first two months of the pandemic, there was approximately a 50% reduction in hospital activity compared to pre-pandemic practice [18]. Furthermore, healthcare workers needed to cope with the added challenge of protecting themselves while providing the highest possible standard of care for patients [19, 20]. Guideline alterations recommended managing all patients as though they are potentially COVID infected, and therefore adhering to universal safety precautions [21]. Regarding surgical specialties, certain centers prioritized surgeries with a high chance of cure while those with uncertain benefit and poor prognosis were avoided [18]. Nevertheless, healthcare systems needed to balance managing COVID-19 risk with other critical illnesses requiring medical attention for diseases such as malignancies [22]. The management of HNC was no exception and faced similar burdens during the pandemic. With respect to COVID-19 outcomes, HNC patients are typically of higher risk due to their age, comorbidities, and male predominance [18].

The interpretation of our study necessitates an understanding of the guideline modifications that were specifically undertaken for HNC patient care at our two tertiary care centers. At our first tertiary care center, henceforth known as Center A, all elective surgeries were stopped at the peak of pandemic waves, allowing only prioritized and emergency surgeries, including oncologic cases, to take place. Accordingly, the limited OR time was managed adequately based on a patient-centered approach. An OR Prioritization Committee was formed and met on a weekly basis to review all surgical requests based on priority: P0 was designated for cases requiring surgery within two weeks, and P1 was designated for cases requiring surgery within four weeks. Priority was determined through a transparent discussion of every case, and the limited OR time was allocated to prioritized patients instead of being allocated to surgeons with block operative time to fill. OTL-HNS clinics were often downsized to 50% capacity and telemedicine was promoted when possible. Furthermore, the clinical activities of non-oncological OTL-HNS physicians were significantly reduced to the point that some of these physicians stopped seeing patients entirely during peaks of pandemic waves. At our second tertiary care center, henceforth known as Center B, OR prioritization was handled in a similar fashion to Center A, including the prioritization of HNC cases throughout the peak of pandemic waves. However, it is worth noting that during the first six weeks of the first pandemic wave, essentially all surgery came to a halt in Center B. Accordingly, several patients were directed towards radiotherapy and/or chemotherapy during this period whereas in a non-pandemic situation, they would not have. Another difference is that the OR prioritization committee in Center B met daily, as opposed to weekly in Center A, to decide which patient cases would be operated the following day.

The results of our study show a significant decrease in the periods between date first seen by OTL-HNS until date first presented at tumor board and the date first seen by OTL-HNS until the start date of treatment, indicating an accelerated HNC cancer workup during the pandemic. As per our covariate analysis, these results were not impacted by tumor site, tumor type, and tumor staging. These results were also not impacted by the total HNC case volume during the pandemic, which remained similar to the pre-pandemic total case volume. We postulate that as hospital care became limited towards routine clinical visits and non-urgent or elective operations, care was diverted to cancer patients whose workups and treatments could not be delayed. Accordingly, the mean total treatment package time for patients receiving surgery only, which was defined as the duration of the hospital stay, was significantly reduced to less than 4 days. The total package time for patients receiving surgery plus adjuvant CXRT, defined from the date of first surgery to end of adjuvant treatment, was also reduced to less than 81 days. The opposite was found for those receiving CXRT only, despite there being no significant changes in the number of patients in the CXRT only and other treatment categories. For this group, total package time was defined from start to end date of CXRT, which increased to over 53 days. The significant differences in total package time for those receiving surgery only, CXRT only and surgery plus adjuvant CXRT were highlighted when adjusting for pertinent covariates, namely all tumor characteristics, all tumor characteristics except for metastasis, and all tumor characteristics except for metastasis and tumor size respectively. The gain in the efficiency of HNC cancer care or cancer care in general in our institutions surely has come at the expense of many patients with non-urgent health care needs and delayed elective surgeries with increasing number of patients in surgical wait list as had been documented by provincial statistics.

Our results show a reduction trend in the periods between symptom onset until start date of treatment, first visit to any doctor until start date of treatment, and the date first biopsied until date of histopathological diagnosis. Other studies have acknowledged the cancellation of non-urgent elective surgeries during the first phase of the pandemic—due to a reduction in the number of available spots in intensive care units—resulting in a reduced waitlist for more urgent procedures [23]. Similarly, the decreased wait time from first biopsy to histopathological diagnosis may have resulted from an overall decreased burden on the pathology departments during the pandemic period. Although many elective operations were delayed or canceled, the treatment of HNC patients could not be safely postponed, as it may be associated with increased morbidity and mortality [24]. Thus, while it was initially thought that cancer patient care would be delayed by the pandemic, the cancellation of non-urgent elective clinics and surgeries by OTL-HNS, as well as other disciplines, at our tertiary care centers had the opposite effect. Consequently, our study suggests that the most influential factor for accelerated HNC patientcare at our centers during the pandemic was likely the transparent model of patient-centered OR prioritization among all medical disciplines involved in the workup of these patients.

Another possible, yet less influential, factor for the reduced wait times in HNC treatment could be the initial reluctance of patients to present at the hospital due to the pandemic. Subsequently, they would present at a more advanced stage of their cancer and require faster and more urgent treatment. Although not significant, our results reflect a trend towards more advanced cancer staging at the time of diagnosis. Other studies have shown that in 2020, patients were more reluctant to go to hospitals despite the severity of their condition, showing significant delays in diagnosis, admission and treatment [22, 25]. A study by Tevetoğlu et al. showed that overall rates of T3/T4 tumors were significantly increased in 2020 [22]. These results are consistent with our study showing an upward trend in advanced cancer presentations, including T3/T4, N1, N2, and those with metastatic disease. It is worth noting that tumor board meetings were never rescheduled or canceled at either of our tertiary care centers, which other studies have suggested as a possible cause for delays in HNC workup and more advanced staging at diagnosis at other hospitals during pandemic waves [25].

As expected, our results show a significant increase in patients who had their first encounter with OTL-HNS through telemedicine during the pandemic. Furthermore, there was a greater tendency for patients to be seen by telemedicine during their first visit with any doctor, including primary care, for index symptoms during the pandemic. With respect to patient preferences, a 2021 study showed that the majority of HNC patients at a tertiary care center preferred in-person surveillance of their condition with a physical exam; however, those who preferred telemedicine cited convenience and the desire to avoid infection [26]. With respect to patient satisfaction, another 2021 study showed that OTL-HNS patients at two tertiary care centers were overall highly satisfied with teleconsultations and believed that its main advantages were earlier care and faster service [10]. Other notable advantages include increased availability of in-person care, decreased travel time/cost for patients, and group decision making with family members and other healthcare providers. At our tertiary care centers, telemedicine allowed OTL-HNS physicians to conduct simple interactions such as revision of test results through virtual appointments, hence freeing up clinic time for in-person appointments that required more active interventions. Although these advantages support the continued implementation of telemedicine, disadvantages such as lack of physical exam, potentially missed diagnoses, and lack of patient access/comfort with technology must be dealt with to increase the long-term efficacy of telemedicine in HNC patient care [10, 26].

Regarding HNC treatment, our study shows no significant changes in the proportion of HNC cases that were treated by surgical versus non-surgical means during the pandemic. Similarly, there were no significant changes in the proportion of cases that were treated with curative versus palliative intent during the pandemic. This indicates that HNC treatment algorithms at our tertiary care centers were not significantly affected by guideline alterations that aimed to re-allocate healthcare workers and equipment, reduce the number of elective surgeries, and increase the availability of hospital beds. A non-significant trend towards decreased treatments of curative intent was observed which can be related to the upward trend in advanced cancer presentations previously discussed. Additionally, the halting of surgeries during the first six weeks of the pandemic by Center B may have played a role in results demonstrating a non-significant downward trend in the number of patients receiving surgery only. It is worth noting that the length of hospitalizations following major HNC surgeries are usually prolonged, which potentially puts HNC patients at risk of coronavirus infection [18]. Accordingly, it was thought that HNC postoperative care would be significantly shortened by pandemic guideline alterations. However, our study demonstrates that there was no significant difference in the length of postoperative hospitalization during the pandemic, meaning that postoperative care of HNC patients was not jeopardized.

### Study limitations

There are several limitations to this study that limit interpretation of the results. It is worth noting that HNC workup was affected by the variability of multiple factors: primary care center or physician on presentation, telemedicine consults, past medical history, tertiary care center for OTL-HNS workup, and supervising OTL-HNS surgeon for each patient. In particular, it is worth noting that each of the two tertiary care centers in this study had minor differences in OR prioritization that dictated changes in HNC patient care during the pandemic. Our conclusions are limited by the timing of our pandemic cohort during the early phase of the COVID-19 pandemic, particularly the first and second waves. Further investigation into later waves of the pandemic may yield differing results as the general population and healthcare system gradually adapted, and their daily practices returned closer to normalcy.

Although multiple worldwide COVID-19 waves have passed at the time of this writing, it is evident that healthcare systems across the globe still face the threat of emerging variants and new potential pandemics in the future. This study only investigated two centers in the same city, thereby limiting the external validity as it relates to other cities and provinces within Canada which may have implemented different triage policies during this time [27]. Further exploration of HNC patient care in other Canadian centers during the pandemic, along with a prospective evaluation of this transparent model for patient-centered OR prioritization in those different settings, would help determine its role on the national level moving forward. The results are also limited in their comparison to other countries which utilize healthcare systems unlike the publicly funded single-payer system in Canada (Medicare), especially in terms of wait times and access to services [28–30]. For example, a study by Schoonbeek et al. in the Netherlands, also found a decrease in the mean time to treat interval during the first wave of the pandemic, which they attributed in part to a decline in overall patient volume and national quality indicators for HNC patient care [31]. The decrease from 37 days in the pre-pandemic to 30 days in the pandemic was smaller, however, as were the mean wait times for both groups when compared to those found in the present study [31]. Together, results of this study along with similar work in different parts of the world have enduring global significance and will inform practice for future outbreaks and for regions with a rising incidence of COVID-19.

## Conclusion

This retrospective study compares HNC patient care in pre-pandemic and pandemic patient cohorts. The outcomes reveal that the duration of the HNC workup in two tertiary care centers, including between the date first seen by OTL-HNS to the starting date of treatment, was significantly reduced during the pandemic. Consequently, a blueprint exists for establishing institutional guidelines that allocate an appropriate level of priority and resources towards HNC care during emergency situations, such as a worldwide pandemic, and increase the efficiency of HNC workup in non-emergency scenarios. The highlight of this blueprint is a transparent model for patient-centered OR prioritization. However, inefficiencies in HNC patient care during non-pandemic times that were highlighted by this study, prolonged duration between being seen by primary care and OTL-HNS during the pandemic, as well as diagnoses that were potentially missed by telemedicine consultations during the pandemic highlight areas of improvement in HNC workup and warrant further investigation.

## Data Availability

The datasets used and/or analyzed during the current study are available from the corresponding author on reasonable request.

## Appendix

See Tables 6, 7.

**Table 6:** Summary of data points for retrospective chart review

Demographics	Age (Yrs) Sex (M/F) Comorbidities (PMHx/PSHx) Smoking (Y/N) Alcohol (Y/N)
Presentation	Date Onset of symptoms (mm-yyyy) Date seen first by any doctor for index symptoms (dd-mm-yyyy) Date referral made to OTL-HNS (dd-mm-yyyy) Date first seen by OTL-HNS (dd-mm-yyyy) Was the first encounter with OTL-HNS by telemedicine or in-person? (Y/N) If the first encounter was by telemedicine, when was the first in-person visit? (dd-mm-yyyy)
Workup	Date first biopsy made (dd-mm-yyyy) Date first histopathologic/cytopathologic diagnosis made (dd-mm-yyyy) Date presented at HNC tumor board (dd-mm-yyyy) Date first imaging—CT, MRI, PET/CT (dd-mm-yyyy)
Tumor Characteristics	Cancer site/subsite Cancer type AJCC stage TNM
Treatment	Tumor board recommendation of treatment Date Start of treatment: Chemo and/or radiotherapy start date, or surgery date if primary surgery (dd-mm-yyyy) Date Adjuvant CTX and/or RTX started, if had surgery first (dd-mm-yyyy) Date surgical pathology reported (dd-mm-yyyy) Did the treatment have curative intent or palliative only? (Y/N) Date CTX and/or RTX completed (dd-mm-yyyy)
Post-Treatment	Postoperative complications Length of postoperative hospital stay Chemo/radiotherapy complications

**Table 7:** Exclusion criteria

1	Patients below the age of 18 on presentation to first OTL-HNS consult
2	First OTL-HNS consult occurred outside of study period (01/07/2019–31/10/2020)
3	Progression of ongoing HNC case (not recurrence) that began prior to 01/07/2019
4	Cases of benign HN mass (no malignancy proven)
5	Five or more dates prior to the date of start of treatment (out of eight total) are missing in the patient chart

## Abbreviations

HNC: Head and Neck Cancer
OR: Operating Room
OTL-HNS: Otolaryngology—Head and Neck Surgery
AJCC: American Joint Committee on Cancer
CXRT: Chemotherapy and/or Radiotherapy
